# Longitudinal Change in Retinal Nerve Fiber Layer Thickness and Intraocular Pressure in Young Adults

**DOI:** 10.1167/tvst.14.4.3

**Published:** 2025-04-01

**Authors:** Samantha Sze-Yee Lee, Gareth Lingham, Alex W. Hewitt, David A. Mackey

**Affiliations:** 1 University of Western Australia, Centre for Ophthalmology and Visual Science (incorporating the Lions Eye Institute), Perth, WA, Australia; 2 Centre for Eye Research Ireland, School of Physics, Clinical and Optometric Sciences, Technological University Dublin, Dublin, Ireland; 3 Centre for Eye Research Australia, University of Melbourne, Royal Victorian Eye and Ear Hospital, Melbourne, VIC, Australia; 4 School of Medicine, Menzies Research Institute Tasmania, University of Tasmania, Hobart, TAS, Australia

**Keywords:** glaucoma, intraocular pressure, retinal nerve fiber layer, the raine study, young adults

## Abstract

**Purpose:**

Age-related changes in glaucoma endophenotypes have been described thoroughly, yet, there are limited data on the normal age-related changes in young adults. This study profiles the 8-year longitudinal change in peripapillary retinal nerve fiber layer (pRNFL), intraocular pressure (IOP), and central corneal thickness (CCT) in young adults.

**Methods:**

A community-based cohort of young adults from the Raine Study underwent eye examinations that included optical coherence tomography of the optic disc, tonometry, and pachymetry when they were 20 and 28 years old. The main outcome measures were the changes in pRNFL thickness, IOP, and CCT over 8 years, adjusted for sex, ethnicity, and other potential confounders.

**Results:**

A total of 693, 712, and 680 participants were included in the pRNFL, IOP, and CCT analyses, respectively. Over the 8 years, the global pRNFL reduced from a mean of 100.6 ± 9.3 to 97.9 ± 9.4 µm, at an average rate of 0.27 µm/year (95% confidence interval [CI], 0.24–0.30). Sectoral pRNFL similarly thinned by 0.06 to 0.38 µm/year, but this thinning was not statistically significant at the superotemporal and inferonasal sectors. IOP decreased and CCT increased between 20 and 28 years old, at an average rate of 0.18 mm Hg/year (95% CI, 0.15–0.20) and 0.18 µm/year (95% CI, 0.10–0.27), respectively.

**Conclusions:**

During the third decade of life, there is a decrease in pRNFL thickness and IOP in healthy adults.

**Translational Relevance:**

The current study findings will enable clinicians to differentiate potential pathological change from normal age-related variations in these measures.

## Introduction

As the human eye ages, several well-documented physiological changes occur. Thickening and hardening of the crystalline lens, loss of retinal ganglion cells (RGCs), and decreases in the number and density of retinal photoreceptors are only a few examples of a myriad of age-related changes that the human eye undergoes.^1^ An acceleration in any of these changes may lead to pathology or early onset of disease. For example, although there is a normal age-related loss of RGCs that may not or only minimally impact visual function, an excessive loss of the RGC axons can result from diseases such as glaucoma.^2^^,^^3^ According to the World Glaucoma Association,^4^ glaucoma is a chronic, progressive, degenerative disorder of the optic nerve that produces visual field damage. Given the progressive nature of the disease and the normal age-related reduction in RGC count, early diagnosis of glaucoma is challenging. Delay in glaucoma diagnosis was identified by Grant and Burke in 1984^5^ as one of three major reasons that people go blind from the disease, and remains a major issue today.^6^

Another important glaucoma phenotype is the intraocular pressure (IOP). Although not a defining feature, raised IOP remains an important and the only modifiable risk factor for glaucoma. However, the age-related variation in IOP remains unclear, with a lack of consensus on whether it tends to increase or decrease with age.^7^^–^^15^

To recognize an abnormally fast rate of change in phenotypic measures of glaucoma, one must first understand the normal rate of age-related change. However, there is limited data on the normative age-related change in IOP or measures of the integrity of the RGC, such as peripapillary retinal nerve fiber layer (pRNFL) thickness, in young adults with healthy eyes. This is particularly important given the increased reliance on commercial optical coherence tomography (OCT) for assessing RGC integrity. OCT devices usually have their own normative databases constructed from data obtained from a few hundred European eyes with a wide age range. For example, Heidelberg Engineering based their reference database on the scans of 330 healthy European individuals ranging from 20 to 90 years of age.^16^ However, their reference population is limited by the small sample spanning a wide age range.

Even though glaucoma tends to affect older adults, glaucoma can occur at any age and we should consider the possibility that subtle subclinical changes, which only later become visually significant, may occur earlier in life. Moreover, the detection of glaucoma in young adults is critical as a younger age of disease onset results in a longer duration of morbidity, loss of productivity in this working-age group, and more time for the disease to progress. Thus, documenting the normal age-related rate of change in ocular parameters in this age group is critical in differentiating the normal or expected changes from the pathological or prepathological ones. To address this goal, we aim to profile the 8-year longitudinal change in pRNFL thickness and IOP in a community-based cohort of young healthy adults. As a supplementary aim, we additionally explored the longitudinal change in CCT that, although not generally considered a phenotypic measure of glaucoma, affects the evaluation of IOP.

## Methods

### Study Sample

Participants comprise the Generation 2 (Gen2) cohort of the Raine Study, which is a multigenerational cohort study that started when more than 2900 pregnant women (Gen1) in Perth, Western Australia, were enrolled into the study during pregnancy in 1989–1991. A total of 2868 offspring were born to these women in 1989–1992, forming the Gen2 cohort. These participants have since been undergoing a series of health and medical examinations, with the most current examinations commencing in 2023, as part of the 33-year follow-up. All examinations have been approved by the University of Western Australia Human Research Ethics Committee and abided by the Declaration of Helsinki. All participants provided written informed consent before each eye examination and after they were given a full explanation of the nature of the study.

### Eye Examination

The Gen2 20-year visit took place in 2010–2012 and included a comprehensive baseline eye examination at the Lions Eye Institute in Western Australia. Participants then returned for a follow-up eye examination in 2018–2020 as part of the Gen2 28-year visit (although the period of the 28-year follow-up was cut short by the coronavirus disease 2019 pandemic, thus reducing the number of participants). The protocols for both eye examinations have been described in detail previously.^17^^,^^18^ The eye examinations at both visits included ocular biometry (IOLMaster V.5; Carl Zeiss Meditec AG, Jena, Germany), and Spectral domain (SD) OCT imaging (Spectralis HRA+OCT; Heidelberg Engineering, Heidelberg, Germany). The SD-OCT was performed after instillation of 1 drop of tropicamide 1% in both eyes to achieve mydriasis, improving imaging quality. before imaging, the central corneal radius was entered into the OCT to account for corneal curvature-related magnification effects. The scan centered and focused on the optic disc. One-hundred frames of a circular B-scan made up of 768 A-scans centered on the optic disc were taken to obtain the pRNFL thickness. Given that the scan diameter was fixed at 12° regardless of scan focus and eye size, the actual scan diameter varied from 3.1–4.3 mm, as estimated by the OCT based on the scan focus and included as a covariable in the models with pRNFL thickness as the outcome measure. Scans with signal-to-noise quality less than 20 were discarded from the analysis.^19^ The 28-year scans were acquired with the 20-year (baseline) scans set as the reference.

Tonometry was conducted using a rebound tonometer (ICare TA01i, ICare Finland Oy, Vantaa, Finland). Central corneal thickness (CCT) was measured using Scheimpflug imaging (Pentacam, Oculus Optikgerate GmbH, Wetzlar, Germany). All tests and imaging were conducted without contact lens wear. The same protocol and device models for SD-OCT imaging, and IOP and CCT measurements were used at both visits. Self-administered questionnaires collected information on contact lens wear and previous laser refractive corneal surgery.

### Statistical Analysis

Analyses were conducted on R version 3.6.2 (The R Foundation for Statistical Computing Platform), and the level of significance was set at *P* < 0.05. Continuous measurements were described in terms of mean and standard deviation or median and interquartile range (IQR) as appropriate. Linear mixed-effect models were used to explore the longitudinal change with age, as it allowed for data from both eyes at all visits to be included in the analysis with a random intercept and slope for participants entered into the models to account for the repeated measure (2 eyes and 2 time points). The suitability of linear mixed-effect models was further confirmed after checking for the normality of the residuals of the models. The main explanatory factor is age, with sex, ethnicity, and current axial length included in the models as co-variates. The pRNFL analysis additionally controlled for Bruch's membrane opening area diameter, scan diameter (in millimeters), and baseline IOP. The longitudinal IOP analysis additionally controlled for CCT, the time of measurement, body mass index, resting heart rate, and systolic blood pressure. The CCT longitudinal analysis also corrected for contact lens wear and excluded participants who had undergone laser refractive surgery or other corneal surgery. The interaction effect of age with each of these factors were explored in the models to identify the factors that affected the longitudinal rate of these phenotypic changes.

## Results

**Figure 1.**
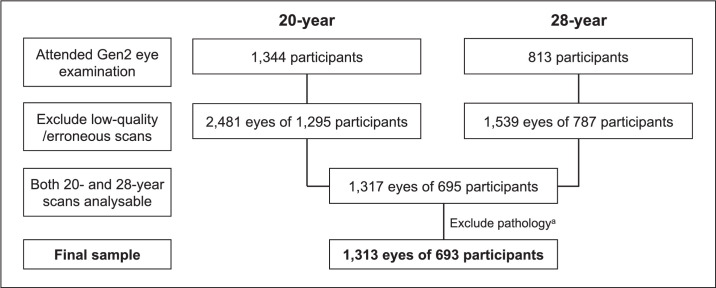
Sample size and exclusions for peripapillary retinal nerve fiber thickness analysis. ^a^Two eyes of one participant with glaucoma and two eyes of one participant with suspected bilateral papilledema were excluded.

A total of 1,344 and 813 participants attended the 20-and 28-year eye examination, respectively. The mean follow-up duration was 8.2 ± 0.5 years, ranging from 6.5 to 10.0 years. For the pRNFL thickness analysis, 1,313 eyes of 693 participants were included after excluding poor quality scans and suspected pathology (Fig. 1). For the IOP analysis, 1,424 eyes of 712 participants had IOP measurements at both visits and were included. After excluding 8 females and 6 males who had laser refractive surgery, the CCT analysis comprise 1,285 eyes of 680 participants. On average, participants were 20.1 ± 0.4 years old at the 20-year visit and 28.2 ± 0.5 years at the 28-year visit. The proportions of males and females as well as distributions of ethnicities were not significantly different between participants who returned and those who did not return for the follow-up. Although those who attended the follow-up were statistically younger at baseline than those who did not (*P* = 0.037), this difference in baseline age was only 0.1 years (∼1.2 months) (Table 1).

**Table 1.** Characteristics of the Raine Study Gen2 Participants Who Did and Did Not Attend the 28-year Follow-up Eye Examination

	Attended	Did Not Attend	*P* Value
Total attendance (n)	736	608	
Female sex (n, %)	375 (48.6%)	277 (45.6%)	0.06
Baseline age (years; mean ± SD)	20.0 ± 0.4	20.1 ± 0.5	0.037^a^
	Range, 18.3–22.1	Range, 19.0–22.1	
Ethnicity (n, %)			0.14
European	638 (86.7%)	510 (83.9%)	
East Asian	19 (2.6%)	11 (1.8%)	
South Asian	12 (1.6%)	9 (1.5%)	
Mixed/other	67 (9.1%)	78 (12.8%)	

SD, standard deviation.

Total attendance at 20- and 28-year *n* = 1344 and 813, respectively; not all participants who attended the 28-year examination had an eye test at 20 years.

a Group difference statistically significant at *P* < 0.05.

### pRNFL Thickness

**Figure 2.**
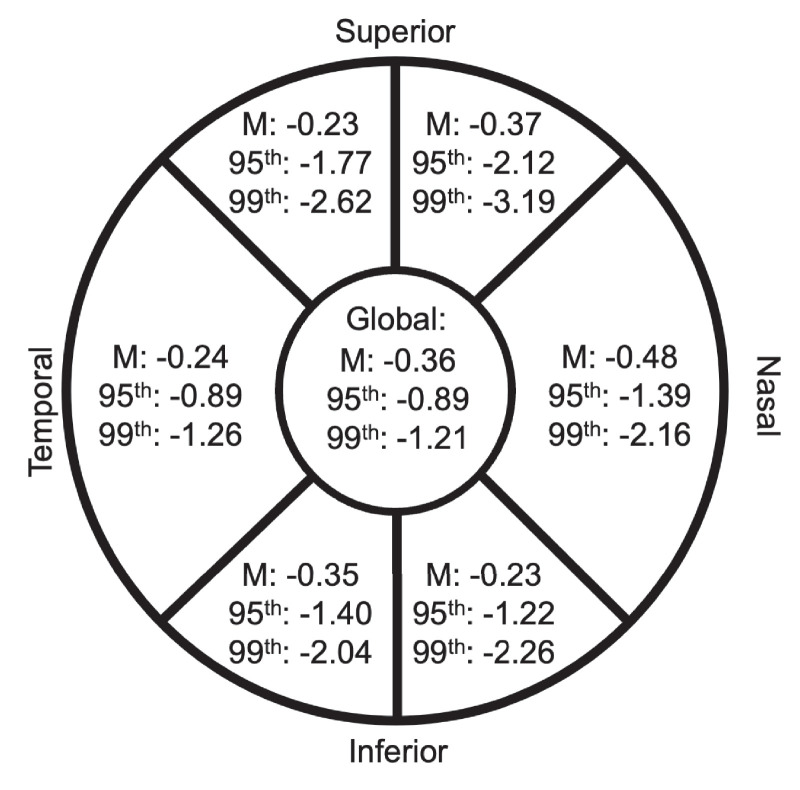
Average rate of change in global and sectoral pRNFL (µm/year), along with the lower 95th and 99th centile cut-offs, between 20 and 28 years old.

On average, the global pRNFL decreased from a mean of 100.6 ± 9.3 µm to 97.9 ± 9.4 µm between 20 and 28 years. Sectoral pRNFL thickness similarly declined over time. This thinning of the pRNFL was statistically significant (Table 2) after adjusting for sex, ethnicity, axial length, baseline IOP, Bruch's membrane opening diameter, and scan diameter. Supplementary Table S1 presents the associations of each covariable with each sector of pRNFL. In unadjusted analyses, Bruch's membrane opening diameter and axial length are each significantly associated with the pRNFL globally and at all sectors (*P* ≤ 0.044). Male sex was associated with thinner pRNFL at the inferotemporal and temporal sectors, and participants of East Asian ethnicity had a thinner pRNFL at the superonasal and nasal sectors. With the exception of axial length, none of these variables had significant interaction effects with age on pRNFL thickness. The 95th and 99th centiles cut-offs in pRNFL rate of change in this healthy sample are additionally shown in Figure 2.

**Table 2.** Longitudinal Change in pRNFL Thickness Between 20 and 28 Years of Age (Expressed as µm/Year)

Sector	Estimate [95% CI]	F-Statistic, *P* Value
Main effects of age—univariable analysis		
Global	−0.33 [−0.36 to −0.31]	F_1,1827_ = −16.3, *P* < 0.001
Superotemporal	−0.19 [−0.29 to −0.09]	F_1,1894_ = −1.6, *P* = 0.12
Inferotemporal	−0.34 [−0.42 to −0.26]	F_1,1883_ = −5.4, *P* < 0.001
Superonasal	−0.45 [−0.56 to −0.35]	F_1,1903_ = −4.5, *P* < 0.001
Inferonasal	−0.21 [−0.30 to −0.12]	F_1,1879_ = −0.8, *P* = 0.43
Temporal	−0.23 [−0.28 to −0.18]	F_1,1893_ = −8.6, *P* < 0.001
Nasal	−0.48 [−0.54 to −0.42]	F_1,1896_ = −9.9, *P* < 0.001
Main effects of age—multivariable analysis^a^		
Global	−0.27 [−0.30 to −0.24]	F_1,2036_ = 293.1, *P* < 0.001
Superotemporal	−0.10 [−0.21 to −0.02]	F_1,2161_ = 5.5, *P* = 0.009
Inferotemporal	−0.27 [−0.36 to −0.18]	F_1,2137_ = 33.1, *P* < 0.001
Superonasal	−0.29 [−0.40 to −0.17]	F_1,2167_ = 24.2, *P* < 0.001
Inferonasal	−0.06 [−0.16 to −0.06]	F_1,2130_ = 4.7, *P* = 0.033
Temporal	−0.26 [−0.32 to −0.21]	F_1,2154_ = 84.0, *P* < 0.001
Nasal	−0.38 [−0.46 to −0.31]	F_1,2153_ = 109.3, *P* < 0.001
Age × axial length interaction effects^b^—multivariable analysis^a^		
Global	0.03 [0.00 to 0.06]	F_1,1893_ = 5.1, *P* = 0.023
Superotemporal	−0.06 [−0.17 to 0.05]	F_1,1896_ = 1.3, *P* = 0.26
Inferotemporal	0.04 [−0.05 to 0.12]	F_1,1894_ = 0.7, *P* = 0.40
Superonasal	0.06 [−0.04 to 0.17]	F_1,1904_ = 1.4, *P* = 0.24
Inferonasal	0.04 [−0.06 to 0.13]	F_1,1897_ = 0.5, *P* = 0.48
Temporal	−0.01 [−0.06 to 0.05]	F_1,1901_ = 0.1, *P* = 0.82
Nasal	0.06 [−0.01 to 0.12]	F_1,1903_ = 2.7, *P* = 0.10

CI, confidence interval.

a Adjusted for sex, ethnicity, axial length, baseline IOP, Bruch's membrane opening diameter, and scan diameter.

b Change in age effect (µm/year) per 1-mm increase in axial length.

The age × axial length interaction effect on global pRNFL is shown in Table 2. Shorter eyes (less myopic) had faster rates of pRNFL thinning, such that for each millimeter shorter in axial length, global pRNFL thinned by a further 0.03 µm/year. This interaction effect was not significant at any of the sectors, although most showed a similar trend.

### Intraocular Pressure

The mean IOP decreased from 15.4 ± 3.4 mm Hg to 13.9 ± 3.4 mm Hg (*P* < 0.001) during the 8 years, at an average rate of 0.18 mm Hg/year. There was a significant CCT × age interaction effect, such that every 10-µm increase in CCT was linked to a decreased rate of IOP change by a further 0.008 mm Hg/year (*P* = 0.004).There was no other significant interaction effect between age and other explanatory variables on IOP (Table 3).

**Table 3.** Associations of IOP in Young Adults^a^

	Estimate [95% CI]	F-Statistic, *P* Value
Main effects		
Follow-up duration (per year)	−0.18 [−0.20 to −0.15]	F_1,1937_ = 261.6, *P* < 0.001
Male sex (ref = female)	−0.09 [−0.43 to 0.25]	F_1,3523_ = 0.3, *P* = 0.60
Axial length (mm)	0.23 [0.08 to 0.39]	F_1,1616_ = 9.0, *P* = 0.003
CCT (µm)	0.03 [0.03 to 0.04]	F_1,2066_ = 259.2, *P* < 0.001
Body mass index	0.01 [−0.01 to 0.03]	F_1,2819_ = 0.7, *P* = 0.39
Systolic BP (mm Hg)	0.02 [0.01 to 0.03]	F_1,3690_ = 7.4, *P* = 0.004
Diastolic BP (mm Hg)	0.01 [−0.00 to 0.03]	F_1,3882_ = 2.2, *P* = 0.13
Resting heart rate	0.00 [−0.02 to 0.01]	F_1,3376_ = 0.2, *P* = 0.64
Interactions with follow-up duration*^b^*		
CCT (µm)	−0.0008 [−0.001 to 0.0002]	F_1,2964_ = 8.3, *P* = 0.004

BP, blood pressure; CCT, central corneal thickness; CI, confidence interval.

a Additionally corrected for ethnicity and time of measurement.

b Only significant interactions shown.

### Central Corneal Thickness

Excluding those with a history of laser refractive surgery, the mean CCT was 537.5 ± 32.5 µm and 540.2 ± 32.6 µm at 20 and 28 years. After accounting for sex, ethnicity, and contact lens wear, CCT thickened at an average rate of 0.18 µm/year (Table 4). Contact lens wear and axial length were not associated significantly with CCT or its rate of change (*P* ≥ 0.12).

**Table 4.** Associations of CCT in Young Adults^a^

	Estimate [95% CI]	F-Statistic, *P* Value
Follow-up duration (per year)	0.18 [0.10 to 0.27]	F_1,2286_ = 19.4, *P* < 0.001
Male sex (ref = females)	2.40 [−2.12 to 6.91]	F_1,831_ = 1.1, *P* = 0.30
Contact lens wear	−1.45 [−8.49 to 5.59]	F_1,864_ = 0.2, *P* = 0.69
Axial length (mm)	−0.51 [−2.09 to 1.07]	F_1,2745_ = 0.4, *P* = 0.52

CCT, central corneal thickness; CI, confidence interval.

a Additionally corrected for ethnicity, there were no significant interaction effects.

## Discussion

There have been several studies reporting the age-related pRNFL thickness variation in older adults, all of which generally agree that the pRNFL starts to thin at least from middle-age. Previous estimates for age-related variations in pRNFL thickness vary widely with heterogenous populations, study designs, analytical methods (including adjustments for potential confounders), and OCT models and imaging protocols used. In older adults aged 40 to 90 years, after accounting for ocular magnification effects, cross-sectional studies reported that the rate of global pRNFL thinning varied from −0.14 to −0.56 µm/year in Europeans^20^^–^^24^ and −0.20 to −0.49 µm/year in Asians.^25^^–^^29^

In other cross-sectional studies where young adults or children were nested within a larger sample of study participants, age-related variations in pRNFL thickness have been reported to be −0.15 to −0.26 µm/year in European populations^22^^,^^30^^,^^31^ and −0.19 to −0.23µm/year in East Asians.^32^ This smaller effect size in studies that included children and young adults suggests that younger age groups have smaller changes in pRFNL thickness.

Regardless of study design and protocol, it is generally agreed that the pRNFL thickness remains mostly unchanged or may even thicken during the first two decades of life.^33^^–^^35^ In a cross-sectional study of 5- to 18-year-olds in East Asia, Qian et al.^35^ reported a positive association between age and pRNFL globally and at the superior and nasal quadrants (+0.16, 0.18, and 0.09 µm/year, respectively), after controlling for refractive error. However, the use of both eyes in each participant without correcting for the within-subject correlation may have overestimated the statistical significance of their findings. One study measured the pRNFL of children (9.1 ± 2.3 years old) longitudinally and failed to find a significant change with age after an average follow-up of 3.3 years,^36^ although this null finding may be in part due to its small sample size (*n* = 8 eyes of 8 children).

The current findings suggest that the pRNFL starts to thins around from the third decade of life. The global pRNFL showed a rate of change by −0.28 µm/year, which is a faster rate of thinning than the reported −0.19 µm/year during the third decade of life by a 3-year longitudinal study in Korea.^37^ Interestingly, in an older and independent group of participants, the same study reported that this rate of change increased to −0.57 µm/year during the fourth decade, and then decreased to −0.48 µm/year during the fifth decade. However, as far as we can tell, the authors did not correct for ocular magnification effects (which may overestimate pRNFL thickness measurements in older individuals)^23^ or refractive error (spherical equivalent range of +3.0 to −6.0 D in the study group), and were further limited by their small sample size of approximately 20 eyes per decade. Importantly, with the exception of axial length, none of the covariables tested had an interaction effect with age on longitudinal pRNFL thickness change. Although males had a thinner pRNFL at some sectors, consistent with previous studies,^20^^,^^24^^,^^26^^,^^28^ the rate of longitudinal change did not differ between sexes. Few studies have evaluated the effect of sex on age-related pRNFL thickness variations. Rougier et al.’s cross-sectional study^20^ of more than 200 older adults similarly did not find an age × sex interaction effect on pRNFL thickness. Further exploration on the factors that influence the longitudinal rate of pRNFL change is warranted.

Reports on age-related IOP variations have been conflicting. A reduction in IOP with age in older adults 40 years old or older, with rates of IOP decline ranging from 0.03 to 0.26 mm Hg/year, has been reported in several cross-sectional and longitudinal studies in East Asia.^7^^–^^11^ This finding is contradictory to cross-sectional studies in other parts of the world, which reported that IOP tends to increase with advancing age.^12^^–^^15^ For example, a longitudinal study of 139 participants in Sweden found a +0.05 mm Hg/year increase between 66 and 87 years of age.^12^ A similar rate of change was reported in a cross-sectional analysis of over 100,000 United Kingdom Biobank participants between the ages of 40 and 69 years old. The Blue Mountains Eye Study noted a trend of increasing IOP from 15.9 to 16.1 mm Hg with age in a cross-sectional cohort of individuals aged 49 to 80 years old.^14^ However, after adjusting for potential confounders such as presence of diabetes and myopia, the age effect was only −0.02 mm Hg/year, which was no longer significant.^14^ A population-based cross-sectional study in Singapore observed an IOP increase of 15.1 to 15.5 mm Hg between 40 and 59 years old, but then decreased from 15.5 to 15.0 mm Hg in the seventh decade of life.^15^

Importantly, these previous studies were all conducted in adults aged older than 40 years of age, when cataracts are common. The associated lens thickening with age-related cataracts could cause an increase in the IOP and may explain the inconsistencies in previous study findings. In a healthy younger cohort, the effect of cataracts is reduced significantly. The current study took advantage of this fact and explored the longitudinal change in IOP during young adulthood. Over the 8 years, we found an average 1.5 mm Hg reduction in IOP. However, the approximately 1.8 mm Hg/year decrease reported in the current study only represents the average rate of change, and the linearity of this change cannot be ascertained as only two measurements per eye were taken 8 years apart. More frequent measurements of IOP in a young cohort could help to explore for differential rates of changes during young adulthood and at what age does IOP start increasing.

The mechanism of this overall slight decline in IOP during younger adulthood is unclear, and our multivariable analysis suggests that this age-related decline is independent of blood pressure and body mass index. Studies have documented slower aqueous outflow and shallower anterior chambers in older adults compared with their young counterparts,^38^^,^^39^ which may be linked to the reported IOP increase in older age. However, there is limited research on the aqueous dynamics in children and younger adults. Our recent analyses in the current Raine Study cohort found that axial elongation^40^ and choroidal thickening^41^ continue into the third decade of life, suggesting that ocular development does not cease before young adulthood. Thus, it is conceivable that the aqueous humor drainage system could increase in efficiency as one transitions from childhood to adulthood. However, this explanation is purely speculative and, given the scarcity of research in the aqueous dynamics of children and young adults, further work exploring the mechanisms underlying IOP decrease at this age is warranted.

A limitation of the current study is that almost one-half of the participants were lost to follow-up. This was partly due to the coronavirus disease 2019 pandemic, which forced early cessation of data collection in 2020. Additionally, longer follow-ups are inherently associated with higher participant attrition rates, with this phenomenon more pronounced in young working adult age groups.^42^ The Australian Longitudinal Study on Women's Health reported a 32% attrition rate within 4 years of follow-up in participants aged 18 to 23 years, compared with 10% to 16% in those aged 45 years and older.^42^ Another potential study limitation is the 8-year gap between the two eye examinations, which did not allow us to explore if there was a differential rate of change between years; for example, if pRNFL thinning only started in the mid or late third decade. There are also limitations to our imaging protocol. Unfortunately, the Bruch's membrane opening–radial circle scan on the SD-OCT glaucoma module was not available at the baseline visit, and pRNFL was imaged using a fixed 12° circle scan. This difference resulted in slight variations in the true size of the circle scans. We have attempted to account for this by including axial length and scan diameter in the pRNFL thickness models, but we acknowledge that this strategy may not fully negate magnification effects. Although we have ensured similar protocols in the two eye examinations, as with any longitudinal study, systematic errors, especially in IOP measurement, cannot be ruled out. Given the limited amount of data in the literature on the normal IOP changes during young adulthood, further studies are warranted to ascertain our findings to exclude the possibility of a false-positive finding owing to systematic measurement errors.

Nonetheless, our study design is advantageous over most other studies. First, the current study comprises a relatively large cohort of community-based young adults who are generally representative of the Western Australian population of the same age.^43^ Other studies^37^^,^^44^ that have explored the longitudinal pRNFL change in this age group typically have small sample sizes, with the young age group only making up a small proportion of their sample or comprise individuals with high myopia.^37^

Second, our study is longitudinal in nature. Some cross-sectional studies sample individuals of a much wider age range—as young as 6 years old up to those in their tenth decade of life^28^^,^^33^^,^^45^—and then assume the average difference between each year or decade as the rate of change in eye measures. Such studies assume that the rate of change in eye measures is linear or constant throughout the decades, which is highly unlikely, given that the age-related rate of change in the different aspects of vision function is known to vary with age.^46^^,^^47^ A second issue with cross-sectional studies is that they ignore possible generational variations. For instance, the prevalence of myopia was once thought to decrease with increasing age,^48^ and it is now well-demonstrated that these differences are largely due a generational effect rather than a true age-related decrease in prevalence.^49^ Generational differences have similarly been noted in nonocular environmental factors, such as a decrease in tobacco smoking,^50^ increase in obesity,^51^ and higher levels of education^52^ with younger generations, all of which have been shown to impact eye health or vision in one or more ways. The discrepancy between longitudinal and cross-sectional was demonstrated by Zhang et al.,^21^ who found a −0.14 µm/year pRNFL thickness change in their longitudinal analysis vs. a −0.21 µm/year change in their cross-sectional analysis using the same cohort. Furthermore, unlike most previous studies, our longitudinal IOP analysis accounted for CCT, which is a known modifier of IOP, and our OCT imaging quality and IOP measurements were not affected by cataracts given the young age of our participants.

### Future Directions and Conclusions

With OCTs becoming more widely available and imaging quality improves, other measures of RGC integrity such as macular ganglion cell and inner plexiform layer thickness and Bruch's membrane opening minimum rim width can now be measured, and have been shown to potentially be more sensitive in detecting and monitoring glaucoma. Thus, mapping the longitudinal changes in such measures are a worthwhile endeavor in future cohort studies comprising young adults. Even though glaucoma in young adults is rare and it may not be feasible to detect prepathological changes to the optic nerve at a young age, such reference data would be invaluable in assessing disease onset and progression in young individuals with high genetic risk of glaucoma. For example, individuals carrying a mutation in their *MYOC*, *OPTN*, or other glaucoma Mendelian genes are likely to have early-onset glaucoma (<40 years of age),^53^ yet, up to one-fifth of individuals with mutations may not have manifest glaucoma, even in older age.^54^

Findings from the current study provide a reference with which the longitudinal change in pRNFL thickness and IOP in young adults could be compared. However, the generalizability of the current findings to those measured by other instrument models other than those used in the current study should be cautioned. Replication of the current study in young adults using other instruments (i.e., OCT models, different IOP or CCT measurement methods) is a worthwhile endeavor to confirm the current study findings and map the instrument-specific change. As we continue to examine the Raine Study Gen2 participants, we will continue to map the longitudinal change in these measures in the middle and later decades of life. Understanding this normal rate of change will enable clinicians to differentiate between normal age-related variations and potential pathological changes.

## Supplementary Material

Supplement 1
