# Supplementary material for: Longitudinal Change in Retinal Nerve Fiber Layer Thickness and Intraocular Pressure in Young Adults
Source: Transl Vis Sci Technol. 2025 Apr 1;14(4):3. doi: 10.1167/tvst.14.4.3 (PMC11967999; doi:10.1167/tvst.14.4.3)
Supplement: Supplement 1 [file tvst-14-4-3_s001.docx]

**Supplementary Table 1**. Associations of the pRNFL at each sector in young adults

|  | **Estimate (95%CI)** | **F-statistic, p-value** |
| --- | --- | --- |
| **Global** |  |  |
| Male sex (ref= female) | -0.81 [-2.13 to 0.51] | F_1,693_ = 1.5, p= 0.23 |
| Ethnicity (ref= European) |  |  |
| - East Asian | 0.85 [-3.38 to 5.08] | F_1,683_ = 0.1, p= 0.69 |
| - Others/mixed | 0.26 [-1.83 to 2.36] |  |
| BMO diameter (mm) | 2.34 [1.54 to 3.13] | F_1,2426_ = 33.1, p< 0.001 |
| Scan diameter (mm) | 2.20 [-1.66 to 6.06] | F_1,2444_ = 1.3, p= 0.26 |
| Axial length (mm) | -1.76 [-2.31 to -1.22] | F_1,2403_ = 40.5, p< 0.001 |
| **Superotemporal** |  |  |
| Male sex (ref= female) | -0.53 [-2.97 to 1.90] | F_1,682_ = 0.2, p= 0.67 |
| Ethnicity (ref= European) |  |  |
| - East Asian | 3.90 [-3.90 to 11.70] | F_1,671_ = 0.6, p= 0.33 |
| - Others/mixed | -0.96 [-4.82 to 2.90] |  |
| BMO diameter (mm) | 3.95 [1.53 to 6.38] | F_1,2030_ = 10.2, p= 0.001 |
| Scan diameter (mm) | 5.12 [-6.71 to 16.96] | F_1,2113_ = 0.7, p= 0.40 |
| Axial length (mm) | -2.60 [-4.06 to -1.14] | F_1,1572_ = 12.1, p= 0.001 |
| **Inferotemporal** |  |  |
| Male sex (ref= female) | -4.00 [-6.61 to -1.40] | F_1,691_ = 9.1, p= 0.003 |
| Ethnicity (ref= European) |  |  |
| - East Asian | 8.22 [-0.13 to 16.57] | F_1,681_ = 2.4, p= 0.05 |
| - Others/mixed | -1.88 [-6.01 to 2.25] |  |
| BMO diameter (mm) | 4.22 [2.07 to 6.36] | F_1,2475_ = 14.9, p< 0.001 |
| Scan diameter (mm) | -0.28 [-10.68 to 10.12] | F_1,2491_ = 0.0, p= 0.96 |
| Axial length (mm) | -0.98 [-1.68 to -0.18] | F_1,1964_ = 5.4, p= 0.016 |
| **Superonasal** |  |  |
| Male sex (ref= female) | -0.10 [-2.89 to 2.69] | F_1,693_ = 0.0, p= 0.94 |
| Ethnicity (ref= European) |  |  |
| - East Asian | 16.08 [7.15 to 25.00] | F_1,683_ = 7.7, p< 0.001 |
| - Others/mixed | 4.38 [-0.03 to 8.80] |  |
| BMO diameter (mm) | 5.54 [2.96 to 8.12] | F_1,2259_ = 17.7, p< 0.001 |
| Scan diameter (mm) | 4.19 [-8.36 to 16.73] | F_1,2320_ = 0.4, p= 0.51 |
| Axial length (mm) | -4.80 [-6.38 to -3.22] | F_1,1745_ = 35.4, p< 0.001 |
| **Inferonasal** |  |  |
| Male sex (ref= female) | 1.34 [-1.85 to 4.54] | F_1,698_ = 0.7, p= 0.41 |
| Ethnicity (ref= European) |  |  |
| - East Asian | -4.05 [-14.29 to 6.19] | F_1,688_ = 1.3, p= 0.44 |
| - Others/mixed | 3.54 [-1.53 to 8.61] |  |
| BMO diameter (mm) | 3.86 [1.32 to 6.39] | F_1,2505_ = 8.9, p= 0.003 |
| Scan diameter (mm) | -10.31 [-22.57 to 1.96] | F_1,2512_ = 2.7, p= 0.10 |
| Axial length (mm) | -5.59 [-7.20 to -3.98] | F_1,2039_ = 46.1, p< 0.001 |
| **Temporal** |  |  |
| Male sex (ref= female) | -2.39 [-3.82 to -0.95] | F_1,695_ = 10.6, p= 0.001 |
| Ethnicity (ref= European) |  |  |
| - East Asian | 2.94 [-1.66 to 7.54] | F_1,685_ = 1.4, p= 0.21 |
| - Others/mixed | -1.21 [-3.48 to 1.07] |  |
| BMO diameter (mm) | 1.19 [0.08 to 2.30] | F_1,2352_ = 4.7, p= 0.044 |
| Scan diameter (mm) | -0.53 [-6.75 to 5.69] | F_1,2389_ = 0.0, p= 0.87 |
| Axial length (mm) | 2.29 [1.49 to 3.08] | F_1,1819_ = 32.0, p< 0.001 |
| **Nasal** |  |  |
| Male sex (ref= female) | 1.16 [-0.79 to 3.11] | F_1,697_ = 1.4, p= 0.25 |
| Ethnicity (ref= European) |  |  |
| - East Asian | -11.53 [-17.80 to -5.27] | F_1,689_ = 6.6, p< 0.001 |
| - Others/mixed | 0.12 [-2.98 to 3.21] |  |
| BMO diameter (mm) | 2.77 [1.09 to 4.45] | F_1,2415_ = 10.4, p= 0.001 |
| Scan diameter (mm) | 1.05 [-7.10 to 9.20] | F_1,2449_ = 0.1, p= 0.80 |
| Axial length (mm) | -3.04 [-4.09 to -1.99] | F_1,1895_ = 32.3, p< 0.001 |

BMO= Bruch’s membrane opening; CI= confidence interval; pRNFL= peripapillary retinal nerve fibre layer
